# Abstract of the Proceedings of the Buffalo Medical Association

**Published:** 1863-09

**Authors:** J. F. Miner

**Affiliations:** Secretary


ART. II.—*Abstract of the Proceedings of the Buffalo Medical Association.*

TUESDAY EVENING, August 4, 1863.

Dr. Samo, Vice President, in the Chair.

Reading of the minutes of the last meeting postponed.

*Voted*, that Dr. S. W. Wetmore and Dr. Thomas M. Johnson be admitted members upon compliance with by-laws.

*Dr. Strong* related cases of infantile Cholera—one case of peculiar interest on account of sudden fatal termination. The child was four months old, and had been sick for three or four days when it was presented to his notice in his office. The next day he was called to see it, and found that it had distinct Cholera Infantum. With close attention for twenty-four hours the more violent symptoms abated. Treatment consisted mainly of alteratives and anodynes. The child had been brought up upon a bottle, since the mother had nursing sore mouth, and it was necessary to wean the child from the breast. The second day on visiting it, he found every symptom greatly improved, the child looking as if it would soon be well; had some hesitancy about visiting the child again, it appeared so nearly recovered.—That night it died. This he learned in the morning, and was never more surprised. Having an interest to learn more fully the circumstances of the case, he visited the parents of the child to learn the history. They told him that the child remained the same as when visited by him, until in the night. There was great noise and excitement occasioned by an Irish Negro fight, and the child woke up suddenly in great fright, and there were copious discharges from the bowels. They commenced to prepare an injection which they had previously been directed to use, and before it was ready for administration the child was dead. Had never heard of a child dying of fright, though it was not unusual in times of cholera to hear of cases where fright had produced the disease, in adults, which rapidly terminated in death. That a child should be thus affected, he regarded as quite remarkable.

*Dr. Wyckoff* spoke of the prevalence of diarrhœa and dysentery. Both of these diseases he thought were more prevalent than common at this season of the year.

*Dr. Smith* reported diarrhœa, with great prostration, as very common. Had attended several cases which required stimulants in connection with alteratives. They seemed to more urgently require stimulation than cases he had been accustomed to see. He also related the particulars of one case which proved fatal, the system appearing depressed beyond the power of remedies to effect.

*Dr. Miner* remarked that Diphtheritic disease was also quite prevalent, and that he had attended whole families affected more or less severely.

In some cases the disease appeared to be wholly a local affection, the tonsils, palate, and fauces being covered by the exudation, and yet the general symptoms of the disease were entirely absent, or if not wholly absent, were yet in so mild form, and of so short duration, as to be properly regarded as absent. Had always regarded dipthheria as a constitutional disease, and had now no ground for changing his opinions; still, diphtheritic sore throat did appear with extensive exudation upon tonsils, fauces, and even within the nasal cavity, and yet was unattended by general symptoms. On the other hand, the general disease with slight, or even without any, local exudation was sometimes observed in quite severe form; and from these two facts he had inferred that removing or curing the local manifestation, was not necessarily removing the disease in the least degree. The cases he had recently treated were mostly of mild type and required no medication. He had generally prescribed chlorate of potassa, mainly for its moral effect.

In one of the German neighborhoods diphtheria had prevailed extensively, and fatally, terminating in death in several cases. In one of these he had been urged by the family, and family physician, to make the operation of tracheotomy, which he did, not however with confidence in its beneficial effects. This patient was seven years old, and had been sick eight or ten days, at least, had not been well for that period, and had been gradually growing worse. Upon the fauces, palate, and tonsils could be distinctly seen the yellow, thick, dry exudation of diphtheria, while it was suffering from the general symptoms of severe membraneous croup. Countenance livid, voice almost absent, pulse rapid, and dyspnœa very great, which became worse after administration of chloroform. The condition of the respiration and circulation became so bad before the trachea was opened that it seemed certain life would become extinct before the operation could be completed. Upon opening the trachea it was found completely filled with false membrane—with, to all appearance, the false membrane of croup, and not the exudation of diphtheria—that is, not the dry adherent friable membrane observed in the fauces and upon the tonsils, in the usual form of diphtheria.

It was tenacious, easily detached and removed entire, with the complete impression of the trachea when first removed, which it however lost after being laid upon paper. Respiration became easy, pulse more natural, beating one hundred and twelve per minute. The child soon partook readily of milk and whiskey, and appeared comparatively comfortable until the next day, when respiration increased, becoming more and more rapid until death, which took place about twenty-four hours after the operation.

In regard to the propriety of this practice he had little to say. So far as his experience or observation extended, there was nothing to favor such operation, and whenever he made it, it was rather with defference to the views and opinions of others, than from any confidence he entertained in its beneficial effects. When surgeons have reported great success in tracheotomy for croup, they have misled us by incorrect diagnosis or mis-statement of fact, calling spasmodic croup, membranous, or excluding fatal cases from the report. The point of interest was the appearance of the membrane in the trachea, and its resemblance of croup, while in the throat it was distinctly diphtheritic. Whatever may be said about the differences in the two diseases, when occurring in the form of the case described, they are practically and actually the same. Specimen cases of diphtheria and croup are sufficiently distinctive, while the two diseases often approach each other, and become the same; and he believed that there was always a near relationship in the character of the exudation in these two forms of disease.

*Dr. Wyckoff* spoke of having seen cases of diphtheritic croup and of the almost uniform fatality of the disease. Expressed distrust in the efficacy of tracheotomy, and thought the cases might as well terminate fatally without, as after it, since it did not afford great relief during the remainder of life.

*Dr. Strong* remarked upon the negligence of physicians after being voted members of the Association in complying with the by-laws.—Many of them never take any further steps towards completing their membership. Thought it improper to vote to membership any who did not desire to become members, and show that desire by signing the Constitution and By-Laws and paying initiation fee.

*Voted* to adjourn to the first Tuesday evening in September.

J. F. Miner, *Secretary.*

